# ﻿Taxonomy and phylogeny of *Sidera* (Hymenochaetales, *Rickenella* clade) from China and North America revealing two new species

**DOI:** 10.3897/mycokeys.96.100743

**Published:** 2023-04-20

**Authors:** Zhan-Bo Liu, Hong-Min Zhou, Hong-Gao Liu, Yuan Yuan

**Affiliations:** 1 School of Ecology and Nature Conservation, Beijing Forestry University, Beijing 100083, China Beijing Forestry University Beijing China; 2 School of Agronomy and Life Sciences, Zhaotong University, Zhaotong 657000, China Zhaotong University Zhaotong China

**Keywords:** Phylogenetic analysis, polypore, wood-rotting fungi

## Abstract

*Sidera*, belonging to the *Rickenella* clade of Hymenochaetales, is a worldwide genus with mostly poroid hymenophore of wood-inhabiting fungi. Two new species in the genus, *Sideraamericana* and *S.borealis*, are described and illustrated from China and North America based on morphological and molecular evidence. They were mainly found growing on rotten wood of *Abies*, *Picea* and *Pinus*. *S.americana* is characterized by annual, resupinate basidiomata with silk sheen when dry, round pores (9–11 per mm), a dimitic hyphal system, and allantoid basidiospores measuring 3.5–4.2 × 1 μm. *S.borealis* is characterized by annual, resupinate basidiomata with cream to pinkish buff dry pore surface, angular pores (6–7 per mm), a dimitic hyphal system, and allantoid basidiospores measuring 3.9–4.1 × 1–1.1 μm. Phylogenetic analysis based on a combined 2-locus dataset [ITS1-5.8S-ITS2 (ITS) + nuclear large subunit RNA (nLSU)] shows that the two species are members of *Sidera*, and they are compared with morphologically similar and phylogenetically related species, respectively. An identification key to 18 accepted species of *Sidera* in worldwide is provided.

## ﻿Introduction

Larsson et al. (2006) used the name ‘*Rickenella* clade’ for species in *Rickenella* Raithelh. and 18 additional genera for the first time. *Athelopsislunata* (Romell ex Bourdot & Galzin) Parmasto [= *Sideralunata* (Romell ex Bourdot & Galzin) K.H. Larss.] is a member of *Rickenella* clade in their phylogenetic analysis of Hymenochaetales. Miettinen and Larsson (2011) established the new genus *Sidera* Miettinen & K.H. Larss. to accommodate *Athelopsislunata*, *Ceriporiopsislowei* Rajchenb. [= *Sideralowei* (Rajchenb.) Miettinen], *Skeletocutislenis* (P. Karst.) Niemelä [= *Sideralenis* (P. Karst.) Miettinen] and *Skeletocutisvulgaris* (Fr.) Niemelä & Y.C. Dai [= *Sideravulgaris* (Fr.) Miettinen], because these four species formed a monophyletic group and didn’t group together with any other species or genera within *Rickenella* clade in their phylogenetic analysis of 5.8S + nLSU. Yu et al. (2021) studied the taxonomic positions of the genera *Resinicium* Parmasto and *Skvortzovia* Bononi & Hjortstam, which belong to *Rickenella* clade. With the much richer sampling available to us, the phylogenetic analyses also prompted us to study the taxonomic position of *Sidera* within the *Rickenella* clade.

*Sidera*, a genus with mostly poroid hymenophore of wood-inhabiting fungi distributed in most continents except Africa (Miettinen and Larsson 2011; Liu et al. 2022; Wu et al. 2022a), is treated as a member of *Rickenella* clade within Hymenochaetales, with *Sideralenis* as the generic type. To date, 16 species are accepted in *Sidera* (Miettinen and Larsson 2011; Du et al. 2019, 2020; Liu et al. 2021, 2022). Seven species of *Sidera* have previously been recorded from China: *S.inflata* Z.B. Liu & Y.C. Dai, *S.minutissima* Y.C. Dai et al., *S.parallela* Dai et al., *S.punctata* Z.B. Liu & Y.C. Dai, *S.roseo-bubalina*Z.B. Liu & Y.C. Dai, *S.salmonea* Z.B. Liu et al., and *S.tibetica* Z.B. Liu et al. (Du et al. 2020; Liu et al. 2021, 2022).

Morphologically, *Sidera* is characterized by resupinate, white to cream or buff, mostly waxy fresh basidiomata, mostly poroid (one hydnoid species) hymenophore, a dimitic or monomitic hyphal system with generative hyphae with clamp connections, the presence of rosette-like crystals, and allantoid to lunate, hyaline, thin-walled basidiospores (Miettinen and Larsson 2011; Liu et al. 2021). Species in the genus cause a white rot.

In this study, we focus on *Sidera* represented by eight resupinate specimens from China, and North America. Phylogenetic analysis based on the ITS and nLSU rDNA sequences is carried out and two new species are described. The current study aims to further explore the species diversity of *Sidera* in the Asia-Pacific region, and more importantly, to confirm the taxonomic position of *Sidera* within the *Rickenella* clade of Hymenochaetales, based on the ITS+nLSU phylogenetic analysis. Morphological characters of all 18 currently accepted species of *Sidera* are summarized in Table 1. Furthermore, an identification key to accepted species is provided in the paper.

**Table 1.** The main characteristics of *Sidera* species. Pore and basidiospore sizes mainly from Liu et al. (2022). New species are shown in bold.

Species	Growing habit	Hymenophore	Hyphal system	Cystidioles	Skeletal hyphae in KOH	Spores shape	Spore dimension (µm)
***S.americana***	**Annual**	**Poroid, 9–11/mm**	**Dimitic**	**Present**	**Almost unchanged**	**Allantoid**	**3.5–4.2 × 1**
***S.borealis***	**Annual**	**Poroid, 6–7/mm**	**Dimitic**	**Present**	**Almost unchanged**	**Allantoid**	**3.9–4.1 × 1–1.1**
*S.inflata*	Annual	Poroid, 9–10/mm	Dimitic	Present	Swollen	Allantoid	3–3.3 × 0.9–1.1
*S.lenis*	Perennial	Poroid, 4–6/mm	Dimitic	Present	Swollen	Allantoid to lunate	3.9–4.9 × 1.5–2
*S.lowei*	Annual	Poroid, 6–8/mm	Monomitic	Present, some branched	–	Allantoid	3.5–5 × 1–1.2
*S.lunata*	Annual	Hydnoid, 8–9/mm	Monomitic	Present	–	Allantoid	2.5–3.8 × 1.6–1.9
*S.malaysiana*	Annual	Poroid, 9–11/mm	Dimitic	Present	Swollen	Lunate	2.9–3.2 × 1–1.2
*S.minutipora*	Annual	Poroid, 5–7/mm	Dimitic	Present	Swollen	Allantoid	3.7–4.3 × 1–1.3
*S.minutissima*	Annual	Poroid, 7–9/mm	Dimitic	Present	Almost unchanged	Allantoid	3.8–4.4 × 0.9–1.3
*S.parallela*	Annual	Poroid, 6–8/mm	Dimitic	Present	Almost unchanged	Lunate	2.8–3.3 × 0.9–1.2
*S.punctata*	Annual	Poroid, 8–9/mm	Monomitic	Absent	–	Allantoid to lunate	3.8–4.8 × 1–1.3
*S.roseo-bubalina*	Annual	Poroid, 6–7/mm	Monomitic	Present	–	Lunate	3.9–4.5 × 0.8–1
*S.salmonea*	Annual	Poroid, 7–9/mm	Dimitic	Present	Almost unchanged	Lunate	3–3.5 × 0.9–1.1
*S.srilankensis*	Annual	Poroid, 6–8/mm	Dimitic	Present	Almost unchanged	Lunate	3.5–4 × 1–1.3
*S.tenuis*	Annual	Poroid, 8–10/mm	Dimitic	Present	Almost unchanged	Allantoid	4.2–5 × 0.8–1
*S.tibetica*	Annual	Poroid, 7–8/mm	Dimitic	Present	Almost unchanged	Lunate	2.9–3.1 × 1–1.1
*S.vesiculosa*	Annual	Poroid, 7–9/mm	Monomitic	Present	–	Allantoid to lunate	2.9–3.7 × 0.6–1
*S.vulgaris*	Perennial	Poroid, 6–8/mm	Dimitic	Present, some branched	Almost unchanged	Allantoid to lunate	2.9–3.6 × 0.9–1.4

## ﻿Materials and methods

### ﻿Morphological studies

Macro-morphological descriptions were based on field notes and dry herbarium specimens. Microscopic measurements and drawings were made from slide preparations of dried tissues stained with Cotton Blue and Melzer’s reagent as described by Dai (2010). Pores were measured by subjectively choosing as straight a line of pores as possible and measuring how many per mm. The following abbreviations are used in the description: CB = Cotton Blue; CB– = acyanophilous in Cotton Blue; IKI = Melzer’s reagent; IKI– = neither amyloid nor dextrinoid in Melzer’s reagent; KOH = 5% potassium hydroxide; n (a/b) = number of spores (a) measured from given number of specimens (b); L = mean spore length (arithmetic average of all the spores); W = mean spore width (arithmetic average of all the spores); and Q = variation in the L/W ratios between the specimens studied. When the variation in spore size is shown, 5% of the measurements were excluded from each end of the range, and these values are shown in parentheses. Special color terms follow Petersen (1996) and then herbarium abbreviations follow Thiers (2018). Voucher specimens from the study were deposited in the herbarium of the Institute of Microbiology, Beijing Forestry University (**BJFC**).

### ﻿DNA extraction,PCR and sequencing

Total genomic DNA was extracted from dried specimens by a CTAB rapid plant genome extraction kit (Aidlab Biotechnologies Company, Limited, Beijing, China) according to the manufacturer’s instructions with some modifications (Li et al. 2014). The ITS regions were amplified with primers ITS4 and ITS5 (White et al. 1990). The nLSU regions were amplified with primers LR0R and LR7 (Vilgalys and Hester 1990).

The polymerase chain reaction (PCR) procedure for ITS was as follows: initial denaturation at 95 °C for 3 min, followed by 35 cycles at 94 °C for 40 sec, 58 °C for 45 sec, and 72 °C for 1 min, and a final extension of 72 °C for 10 min. The PCR procedure for nLSU was as follows: initial denaturation at 94 °C for 1 min, followed by 35 cycles at 94 °C for 30 sec, 48 °C for 1 min, and 72 °C for 1.5 min, and a final extension of 72 °C for 10 min (Zhao et al. 2015). Aliquots of PCR products were examined on 2% agarose gels stained with GelStar Nucleic Acid Gel Stain (Lonza Rockland, Inc., Rockland, YN, USA) and examined under UV light. The sequencing of the PCR products was conducted by the Beijing Genomics Institute, Beijing, China, with the same primers used in the PCR reactions. Species were identified by sequence comparison with accessions in the NCBI databases using the BLAST program.

### ﻿Phylogenetic analyses

Phylogenetic trees were constructed using ITS + nLSU rDNA sequences, and phylogenetic analyses were performed with the Maximum Likelihood (ML), Maximum Parsimony (MP) and Bayesian Inference (BI) methods. Sequences of the species and strains were primarily adopted from ITS-based and 28S-based tree topology as described by Miettinen and Larsson (2011) and Liu et al. (2022). New sequences generated in this study, along with reference sequences retrieved from GenBank (Table 2), were aligned by MAFFT 7 (Katoh et al. 2019; http://mafft.cbrc.jp/alignment/server/) using the “G-INS-i” strategy and manually adjusted in BioEdit v.7.2.5 (Hall 1999). Unreliably aligned sections were removed before the analyses, and efforts were made to manually inspect and improve the alignment. The data matrix was edited in Mesquite v3.70 (Maddison and Maddison 2021; https://www.mesquiteproject.org/). The sequence alignment was deposited at TreeBase. Sequences of *Exidiacandida* Lloyd and *Exidiopsiscalcea* (Pers.) K. Wells outside Hymenochaetales obtained from GenBank were used as outgroups to root the tree in the ITS + nLSU analysis.

**Table 2.** Information for the sequences used in this study. * Newly generated sequences for this study. New species are shown in bold.

Species	Specimen no.	Locality	GenBank accession no.	
			ITS	nLSU
*Athelodermamirabile*	TAA 169235	Estonia	DQ873592	DQ873592
*Contumycesrosella*	Redhead 7501	–	U66452	U66452
*Cyphellostereumlaeve*	JJ 020909	Sweden	EU118621	EU118621
*Exidiacandida*	VS 8588	Russia	KY801871	KY801896
*Exidiopsiscalcea*	MW 331	Canada	AF291280	AF291326
*Globuliciumhiemale*	KHL 961221	Sweden	EU118626	EU118626
*G.hiemale*	Hjm 19007	Sweden	DQ873595	DQ873595
*Hyphodermacapitatum*	KHL 8464	Sweden	DQ677491	DQ677491
*H.orphanellum*	NH 12208	Russia	DQ677500	DQ677500
*Odonticiumromellii*	KHL 1514b	Norway	DQ873639	DQ873639
*Peniophorellapraetermissa*	KHL 13164	Estonia	DQ873597	DQ873597
*P.tsugae*	NH 7473	Sweden	–	DQ677505
*Repetobasidiumconicum*	KHL 12338	USA	DQ873647	DQ873647
*Resiniciumaustroasianum*	LWZ 20180417-5	Malaysia	MW414504	MW414450
*R.bicolor*	Miettinen 14049	Finland	MF319079	MF318936
*R.chiricahuaense*	JLL-14605	Canada	–	DQ863692
*R.confertum*	FP-102863	USA	DQ826538	–
*R.friabile*	CBS 126043	New Zealand	MH864058	MH875513
*R.grandisporum*	GGGUY13-008	French Guiana	KY995325	–
*R.lateastrocystidium*	LWZ 20180414-15	Malaysia	MW414509	MW414455
*R.monticola*	FP-150360	Jamaica	DQ826552	DQ863697
*R.mutabile*	FP-102989	Puerto Rico	DQ826556	DQ863699
*R.rimulosum*	FP-150328	Jamaica	DQ826546	–
*R.saccharicola*	FP-102754	Puerto Rico	DQ826547	DQ863691
*R.tenue*	FP-150354	Jamaica	DQ826539	–
*R.* sp.	LWZ 20171015-31	Vietnam	MW414511	MW414457
*Rickenellafibula*	P. Salo 1882		MF319088	–
*R.mellea*	Lamoure 74	–	U66438	U66438
***Sideraamericana***	**Dai 19173**	**Canada**	MW198477*	MW192005*
	**Dai 12730**	**USA**	MW198478*	–
***S.borealis***	**Dai 22822**	**China**	OM974254*	OM974246*
	**Dai 24120**	**China**	OQ134533*	–
	**Cui 11216**	**China**	MW198485*	–
	**Dai 23962**	**China**	OQ134534*	–
	**Dai 23803**	**China**	OQ134535*	–
	**Dai 24187**	**China**	OQ134536*	OQ134528*
	**Dai 23960**	**China**	OQ134537*	–
*S.inflata*	Cui 13610	China	MW198480	–
*S.lenis*	Miettinen 11036	Finland	FN907914	FN907914
	Dai 22834	China	OQ134538*	OQ134529*
	Dai 22854	China	OQ134539*	OQ134530*
*S.lowei*	Miettinen X419	Venezuela	FN907917	FN907917
	Miettinen X426	New Zealand	FN907919	FN907919
*S.lunata*	JS 15063	Norway	DQ873593	DQ873593
*S.malaysiana*	Dai 18570	Malaysia	MW198481	MW192007
*S.minutipora*	Gates FF257	Australia	FN907922	FN907922
	Cui 16720	Australia	MN621349	MN621348
*S.minutissima*	Dai 19529	Sri Lanka	MN621352	MN621350
	Dai 22495	China	OM974248	OM974240
	Dai 18471A	China	MW198482	MW192008
*S.parallela*	Dai 22038	China	MW477793	MW474964
*S.parallela*	Cui 10346	China	MK346145	–
	Cui 10361	China	MK346144	–
	Dai 22635	China	OQ134540*	OQ134531*
*S.punctata*	Dai 22119	China	MW418438	MW418437
*S.roseo-bubalina*	Dai 11277	China	MW198483	–
*S.salmonea*	Dai 23343	China	OM974249	OM974241
	Dai 23354	China	OM974250	OM974242
	Dai 23428	China	OM974251	OM974243
	Dai 23612	China	–	OM974247
*S.* sp.	Dollinger 922	USA	KY264044	–
*S.srilankensis*	Dai 19581	Sri Lanka	MN621345	MN621347
	Dai 19654	Sri Lanka	MN621344	MN621346
*S.tibetica*	Dai 23407	China	OM974252	OM974244
	Dai 23648	China	OM974253	OM974245
	Dai 21057	Belarus	MW198484*	MW192009*
	Dai 22151	China	MW477794*	MW474965*
*S.tenuis*	Dai 18697	Australia	MK331865	MK331867
	Dai 18698	Australia	MK331866	MK331868
*S.vesiculosa*	Dai 17835	Singapore	MH636565	MH636567
	Dai 17845	Singapore	MH636564	MH636566
*S.vulgaris*	Ryvarden 37198	New Zealand	FN907918	FN907918
*Skvortzoviadabieshanensis*	LWZ 20201012-22	China	MW414512	MW414458
*S.furfuracea*	KHL 11738	Finland	DQ873648	DQ873648
*S.furfurella*	KHL 10180	Puerto Rico	DQ873649	DQ873649
*S.georgica*	KHL 12019	Norway	DQ873645	DQ873645
*S.meridionalis*	FP-150236	–	–	AY293197
*S.pinicola*	KHL 12224	USA	DQ873637	DQ873637
*S.qilianensis*	LWZ 20180904-16	China	MW414518	MW414464
*Skvortzoviellalenis*	LWZ 20180921-7	China	MW414521	MW414467
	LWZ 20180921-17	China	MW414522	MW414468

Maximum Parsimony analysis was applied to the ITS + nLSU dataset sequences. The approaches to phylogenetic analysis utilized those conducted by Liu et al. (2022), and the tree was constructed using PAUP* version 4.0 beta 10 (Swofford 2002). All the characters were equally weighted, and gaps were treated as missing data. Trees were inferred using the heuristic search option with tree bisection and reconnection (TBR) branch swapping, and 1000 random sequence addition maxtrees were set to 5000. Branches of zero length were collapsed, and all the parsimonious trees were saved. Clade robustness was assessed using a bootstrap (BT) analysis with 1000 replicates (Felsenstein 1985). Descriptive tree statistics, including the Consistency Index (CI), Homoplasy Index (HI), Rescaled Consistency index (RC), Retention Index (RI), and tree length (TL), were calculated for each Maximum Parsimonious Tree (MPT) generated.

The research using ML was conducted using RAxML-HPC v.8.2.3 (Stamatakis 2014) and RAxML-HPC through the CIPRES Science Gateway V. 3.3 (Miller et al. 2010; http://www.phylo.org). Statistical support values (BS) were obtained using nonparametric bootstrapping with 1000 replicates. The BI analysis was performed with MrBayes 3.2.7a (Ronquist et al. 2012). Four Markov chains were run for two runs from random starting trees for 5 million generations until the split deviation frequency value < 0.01, and the trees were sampled at every 1000 generation. The first 25% of the sampled trees were discarded as burn-in, and the remaining ones were used to reconstruct a majority rule consensus tree and calculate the Bayesian Posterior Probabilities (BPP) of the clades.

A total of 24 models of evolution was scored using PAUP* version 4.0 beta 10 (Swofford 2002). Optimal substitution models for the combined dataset were then determined using the Akaike Information Criterion (AIC) implemented in MrModeltest 2.3 (Posada and Crandall 1998; Nylander 2004). The model GTR + I + G was selected for use in the Maximum Likelihood (ML) and Bayesian Inference (BI) analyses.

Branches that received bootstrap support for Maximum Likelihood (BS), Maximum Parsimony (BP), and Bayesian Posterior Probabilities (BPP) > 70% (BS), 50% (BP), and 0.95 (BPP) were considered to be significantly supported. In addition, the ML analysis resulted in the best tree, and only the ML tree is shown along with the support values from the MP and BI analyses. FigTree v1.4.4 (Rambaut 2018) was used to visualize the resulting tree.

## ﻿Results

The concatenated ITS+nLSU dataset contained sequences from 81 fungal specimens representing 18 *Sidera* taxa (Table 2). The dataset had an aligned length of 2313 characters, of which 1218 were constant, 269 were variable but parsimony-uninformative, and 826 were parsimony-informative. MP analysis yielded three equally parsimonious trees (TL = 5471, CI = 0.369, RI = 0.694, RC = 0.256, HI = 0.631). And the average standard deviation of split frequencies was 0.009886 (BI).

**Figure 1.**
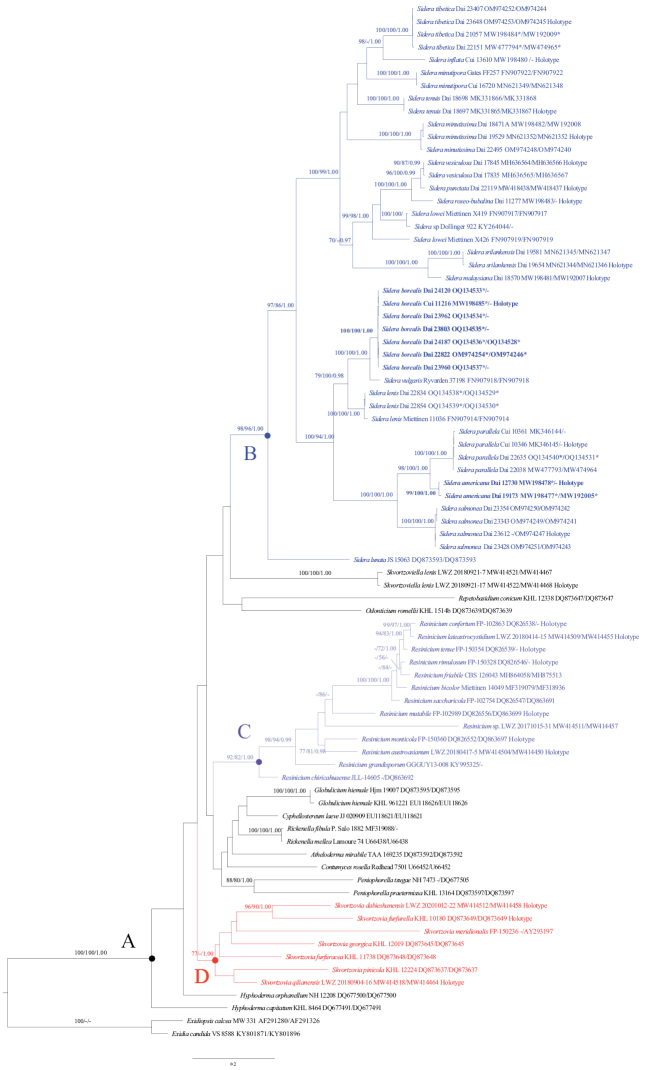
Phylogeny of *Sidera* and other genera in the *Rickenella* clade generated by ML analyses based on combined ITS+nLSU sequences **A** the *Rickenella* clade **B** the genus *Sidera* in modena **C** the genus *Resinicium* in purple **D** the genus *Skvortzovia* in red. Branches are labelled with Maximum Likelihood bootstrap > 70%, parsimony bootstrap proportions > 50%, and Bayesian Posterior Probabilities > 0.95, respectively. New species are indicated in bold. * Newly generated sequences for this study.

The phylogeny (Fig. 1) inferred from the ITS + nLSU sequences confirmed the taxonomic position of *Sidera* (Fig. 1B), *Resinicium* (Fig. 1C), and *Skvortzovia* (Fig. 1D) within the *Rickenella* clade (Fig. 1A) of Hymenochaetales. Species in *Sidera* clustered together with strong support (98% BS, 96% BP, 1.00 BPP) and new species *Sideraamericana* and *S.borealis* clustered in the *Sidera* clade. *S.americana* grouped with *S.parallela* with strong support (98% BS, 100% BP, 1.00 BPP). *S.borealis* grouped with *S.vulgaris* with strong support (100% BS, 100% BP, 1.00 BPP).

Besides, we collected two *Sideralenis* on rotten wood of *Picea* in Yunnan Province, China: Dai 22834 (BJFC 037407) and Dai 22854 (BJFC 037427). This is the first time the species has been reported in China. We have uploaded ITS and nLSU sequences of the two specimens to GenBank (https://www.ncbi.nlm.nih.gov/genbank/) and added them to our phylogenetic analysis (Fig. 1).

### ﻿Taxonomy

#### ﻿ Sidera americana

Taxon classificationFungiHymenochaetalesRickenellaceae

Z.B. Liu & Yuan Yuan sp. nov.

40F875C7-A56A-590A-9A48-17645AFC29B5

MycoBank No: 838379

Figs 2 , 3

##### Diagnosis.

*Sideraamericana* is characterized by annual, resupinate basidiomata with silk sheen when dry, round pores (9–11 per mm), a dimitic hyphal system, and allantoid basidiospores measuring 3.5–4.2 × 1 μm.

**Figure 2.**
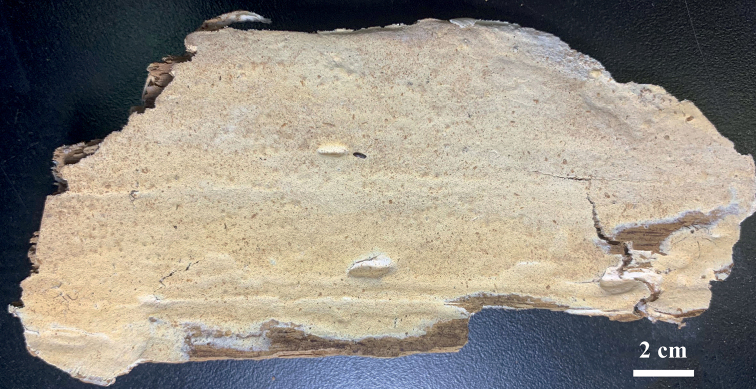
Basidiomata of *Sideraamericana* (Holotype, Dai 12730). Photo by: Zhan-Bo Liu.

##### Holotype.

USA. Connecticut, New Haven, West Rock Park, on rotten stump of *Pinus*, 15.VII.2012, Dai 12730 (BJFC 013037, isotype in CFMR).

**Figure 3.**
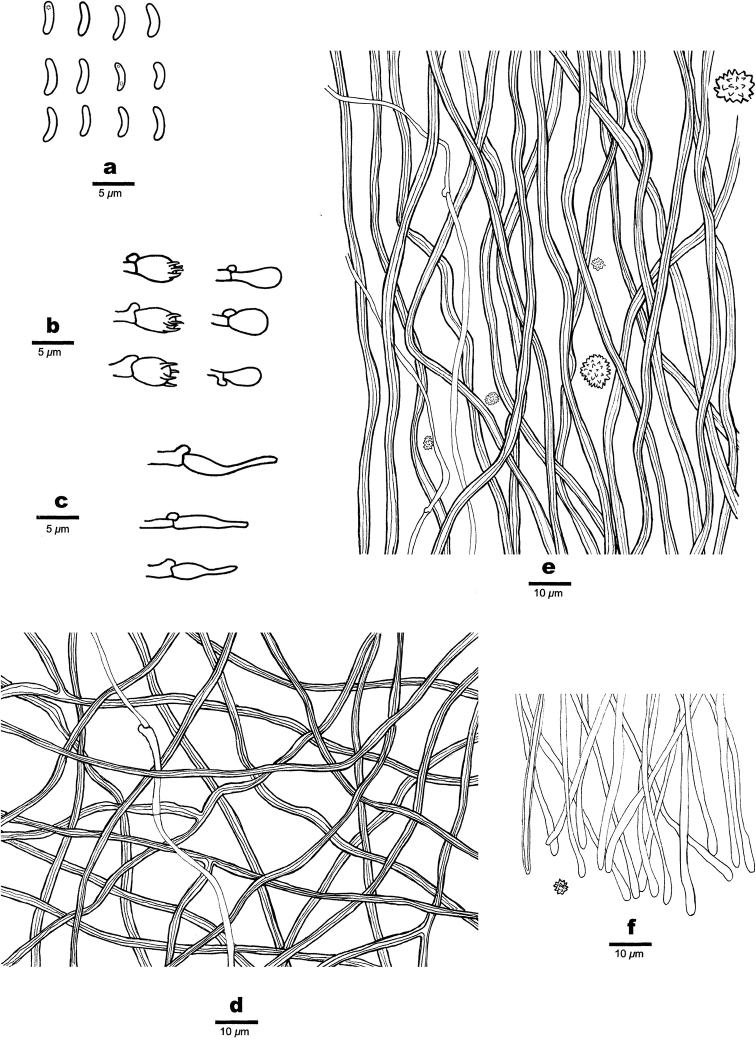
Microscopic structures of *Sideraamericana* (Holotype, Dai 12730) **a** basidiospores **b** basidia and basidioles **c** cystidioles **d** hyphae from subiculum **e** hyphae from trama **f** hyphae at dissepiment edge. Drawings by: Hong-Min Zhou.

##### Etymology.

*Americana* (Lat.): referring to the species occurring in North America.

##### Basidiomata.

Annual, resupinate, soft and without odor or taste when fresh, soft corky when dry, up to 14 cm long, 6 cm wide, and approximately 2 mm thick at center; pore surface white when fresh, becoming cream to buff with silk sheen when dry; sterile margin indistinct; pores round, 9–11 per mm; dissepiments thin, lacerate; subiculum very thin to almost absent; tubes concolorous with poroid surface, up to 2 mm long.

##### Hyphal structure.

Hyphal system dimitic; generative hyphae with clamp connections; skeletal hyphae dominant; all hyphae IKI–, CB–; tissue unchanged in KOH.

##### Subiculum.

Generative hyphae hyaline, thin-walled, unbranched, 1–2.5 μm in diam; skeletal hyphae dominant, thick-walled with a wide lumen, frequently branched, flexuous, interwoven, 2–3 μm diam.

##### Tubes.

Generative hyphae hyaline, thin-walled, unbranched, 1–2 μm in diam, dominating at dissepiment edges; skeletal hyphae dominant in tube trama except dissepiment edges, thick-walled with a wide lumen, unbranched, flexuous, interwoven, 2–3 μm diam; rosette-like crystals abundant, 3–12.5 μm in diam; cystidia absent; cystidioles present, fusoid, hyaline, thin-walled, basally swollen, with a sharp or often hyphoid neck, 13.4–15 × 3.2–4 μm; basidia barrel-shaped, hyaline, bearing four sterigmata and with a basal clamp connection, 6–7 × 3–4.2 μm; basidioles in shape similar to basidia, but slightly shorter.

##### Spores.

Basidiospores allantoid, hyaline, thin-walled, smooth, occasionally with one or two guttules, IKI–, CB–, (3.2–)3.5–4.2(–5) × 1(–1.3) μm, L = 4 μm, W = 1.04 μm, Q = 3.74–3.96 (n = 60/2).

##### Additional specimen examined.

Canada, Ontario, Hamilton, McMaster University, Botanical Garden, on rotten angiosperm wood, 18–20.VII.2017, Dai 19173 (BJFC 027641).

#### ﻿ Sidera borealis

Taxon classificationFungiHymenochaetalesRickenellaceae

Z.B. Liu & Yuan Yuan sp. nov.

8418DA5A-1F8A-5C01-B63C-7D51D5CC0985

MycoBank No: 838385

Figs 4 , 5

##### Diagnosis.

*Sideraborealis* is characterized by annual, resupinate basidiomata with cream to pinkish buff dry pore surface, angular pores (6–7 per mm), a dimitic hyphal system, and allantoid basidiospores measuring 3.9–4.1 × 1–1.1 μm.

**Figure 4.**
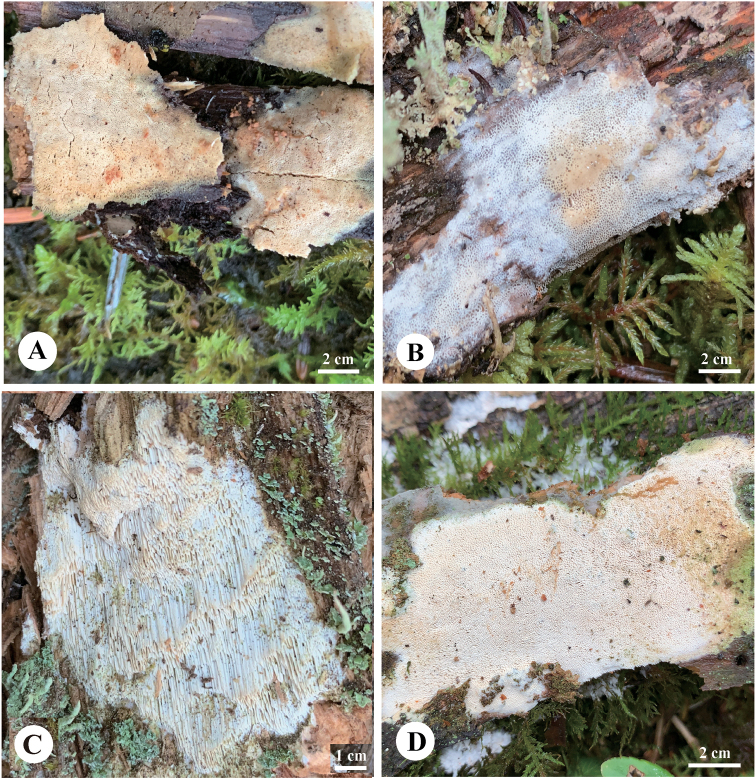
Basidiomata of *Sideraborealis* (Paratypes) **A** Dai 24120 **B** Dai 22822 **C** Dai 23960 **D** Dai 24187. Photo by: Yu-Cheng Dai.

##### Holotype.

China, Shannxi Province, Zhashui County, Niubeiliang Forest Park, on fallen angiosperm trunk, 16.IX.2013, Cui 11216 (BJFC 015331).

**Figure 5.**
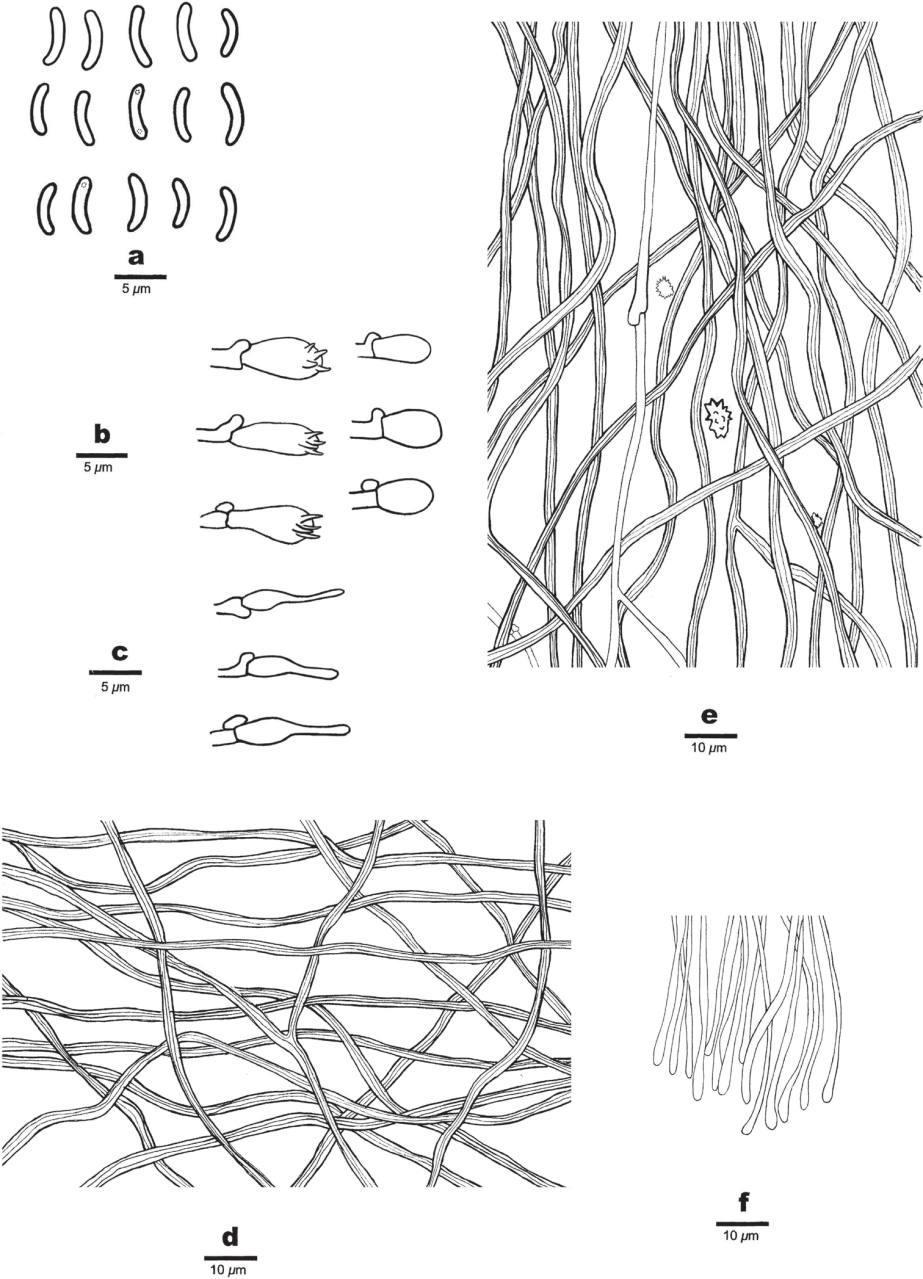
Microscopic structures of *Sideraborealis* (Holotype, Cui 11216) **a** basidiospores **b** basidia and basidioles **c** cystidioles **d** hyphae from subiculum **e** hyphae from trama **f** hyphae at dissepiment edge. Drawings by: Hong-Min Zhou.

##### Etymology.

*Borealis* (Lat.): referring to the species occurring in boreal areas of China.

##### Basidiomata.

Annual, resupinate, soft corky and without odor or taste when fresh, corky when dry, up to 5 cm long, 2 cm wide, and less than 1 mm thick at center; pore surface white to cream or pale buff when fresh, becoming cream to pinkish buff when dry; sterile margin indistinct, white, cottony, thinning out; pores angular, 6–7 per mm; dissepiments thin, entire; subiculum very thin to almost absent; tubes concolorous with poroid surface, less than 1 mm long.

##### Hyphal structure.

Hyphal system dimitic; generative hyphae with clamp connections; skeletal hyphae dominant; all hyphae IKI–, CB–; tissue unchanged in KOH.

##### Subiculum.

Generative hyphae hyaline infrequent, thin-walled, occasionally branched, 1–2 μm in diam; skeletal hyphae dominant, thick-walled with a narrow to medium lumen, occasionally branched, flexuous, interwoven, 1–3 μm diam.

##### Tubes.

Generative hyphae hyaline occasionally present, thin-walled, rarely branched, 1–2 μm in diam, dominating at dissepiment edges; skeletal hyphae thick-walled with a narrow to wide lumen, occasionally branched, flexuous, interwoven, 1–3 μm diam; rosette-like crystals present, 3–6 μm in diam; cystidia absent; cystidioles present, fusoid, hyaline, thin-walled, basally swollen, with a sharp or often hyphoid neck, 17–19 × 2.5–3 μm; basidia barrel-shaped, hyaline, bearing four sterigmata and with a basal clamp connection, 7–8 × 3.5–4 μm; basidioles in shape similar to basidia, but slightly shorter.

##### Spores.

Basidiospores allantoid, hyaline, thin-walled, smooth, occasionally with one or two guttules, IKI–, CB–, (3.5–)3.9–4.1(–4.2) × (0.8–)1–1.1(–1.4) μm, L = 4.01 μm, W = 1.06 μm, Q = 3.78 (n = 60/1).

##### Additional specimens examined.

China, Gansu Province, Zhuoni County, Yaohe Nature Reserve, on rotten wood of *Abies*, 19.VIII.2022, Dai 24187 (BJFC 039430); on rotten wood of *Picea*, 18.VIII.2022, Dai 24120 (BJFC 039364); Jilin Province, Antu County, Dongfanghong Forest Farm, on rotten wood of *Pinus*, 25.VII.2022, Dai 23803 (BJFC 039047); Qinghai Province, Nangqian County, Baizha Forest Farm, on rotten wood of *Picea*, 7.VIII.2022, Dai 23960 (BJFC 039204); Dai 23962 (BJFC 039206); Yunnan Province, Deqin County, Baimaxueshan Nature Reserve, on rotten wood of *Picea*, 5.IX.2021, Dai 22822 (BJFC 037395).

#### ﻿ Sidera tibetica

Taxon classificationFungiHymenochaetalesRickenellaceae

Z.B. Liu, Jian Yu & F. Wu, Journal of Fungi 8: 7 (2022)

461091B8-F86A-55EE-B162-E1FD2ED3620F

Fig. 6

##### Description.

See Liu et al. (2022). Liu et al. (2022) described *Sideratibetica* as a new species based on Tibetan specimens and a photo of holotype. Subsequently, more specimens of the species from Belarus and China (Guangxi, Yunnan and Zhejiang) were collected and we took many photos of the fungus at different stages of growth on different hosts to make it easier for taxonomists to recognize the fungus in the field.

**Figure 6.**
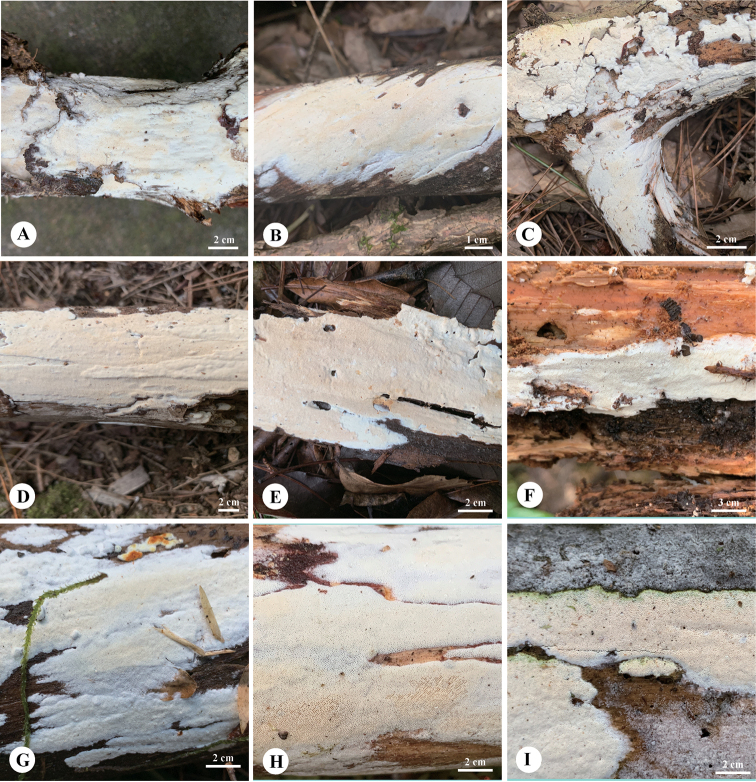
Basidiomata of *Sideratibetica***A** Dai 22321 **B** Dai 22639 **C** Dai 22663 **D** Dai 22151 **E** Dai 20342 **F** Dai 23407 **G** Dai 23486 **H** Dai 23648 (Holotype) **I** Dai 21057. Photo by: Yu-Cheng Dai.

##### Materials studied.

Belarus, Brestskaya Voblasts, Belavezhskaya Pushcha National Park, on rotten wood of *Picea*, 19.X.2019, Dai 21057 (BJFC 032716, paratype). China, Guangxi, Guiping County, Xishan Forest Park, on rotten wood of *Pinus*, 25.XII.2020, Dai 22151 (BJFC 036043, paratype); Xizang, Bomi County, Gangcun Spruce Park, on a rotten branch of *Pinusarmandii*, 27.X.2021, Dai 23648 (BJFC 038220, holotype); Yigong, on a rotten branch of *Pinusarmandii*, 24.X.2021, Dai 23407 (BJFC 037979, paratype); on rotten wood of *Pinusyunnanensis*, 24.X.2021, Dai 23486 (BJFC 038058, paratype); Yunnan Province, Jianchuan County, Yinhe Mountain, on fallen trunk of *Pinus*, 27.IX.2021, Dai 23097 (BJFC 037668, paratype), 28.IX.2021, Dai 23121 (BJFC037692, paratype); on fallen branch of *Pinus*, 27.IX.2021, Dai 23106 (BJFC037677, paratype). Mouding County, Huafoshan Nature Reserve, on rotten wood of *Pinusyunnanensis*, 31.VIII.2021, Dai 22639 (BJFC 037213, paratype); Dai 22663 (BJFC 037237, paratype); on rotten wood of *Pinus*, 31.VIII.2021, Dai 22667 (BJFC 037241, paratype). Wuding County, Shizishan Nature Reserve, on fallen angiosperm trunk, 15.VIII.2019, Dai 20326 (BJFC 031994, paratype); on rotten wood of *Pinus*, 15.VIII.2019, Dai 20342 (BJFC 032010, paratype); Zhejiang Province, Pingyang County, Nanyandangshan Forest Park, on rotten wood of *Pinus*, 3.VI.2021, Dai 22321 (BJFC 036909, paratype).

### ﻿A key to accepted species of *Sidera* in worldwide

**Table:** 

1	Hymenium grandinioid to odontioid	***S.lunata***
–	Hymenium poroid	**2**
2	Hyphal system monomitic	**3**
–	Hyphal system dimitic	**6**
3	Basidiospores mostly < 1 μm in width	**4**
–	Basidiospores mostly > 1 μm in width	**5**
4	Pores 7–9 per mm; basidiospores 2.9–3.7 μm long	***S.vesiculosa***
–	Pores 6–7 per mm; basidiospores 3.9–4.5 μm long	***S.roseo-bubalina***
5	Pores 6–8 per mm; cystidioles present, some branched	***S.lowei***
–	Pores 8–9 per mm; cystidioles absent	***S.punctata***
6	Basidiospores > 1.5 μm in width	***S.lenis***
–	Basidiospores < 1.5 μm in width	**7**
7	Skeletal hyphae becoming swollen in KOH	**8**
–	Skeletal hyphae almost unchanged in KOH	**10**
8	Pores 5–7 per mm; basidiospores 3.7–4.3 μm long	***S.minutipora***
–	Pores 9–11 per mm; basidiospores 2.9–3.3 μm long	**9**
9	Basidiospores allantoid, skeletal hyphae distinctly swollen in KOH	***S.inflata***
–	Basidiospores lunate, skeletal hyphae slightly swollen in KOH	***S.malaysiana***
10	Tramal hyphae parallel along the tubes	***S.parallela***
–	Tramal hyphae interwoven	**11**
11	Generative hyphae at dissepiments even	**12**
–	Generative hyphae at dissepiments with swollen tips	**16**
12	Basidiospores > 3.5 μm long	**13**
–	Basidiospores < 3.5 μm long	**15**
13	Pores < 9 per mm	***S.americana***
–	Pores > 9 per mm	**14**
14	Skeletal hyphae occasionally branched in subiculum and tube trama	***S.borealis***
–	Skeletal hyphae unbranched in subiculum and tube trama	***S.srilankensis***
15	Sterile margin distinct, white; basidiospore length/width > 3	***S.salmonea***
–	Sterile margin indistinct to almost absent; basidiospore length/width < 3	***S.tibetica***
16	Basidiospores < 3.6 μm long	***S.vulgaris***
–	Basidiospores > 3.8 μm long	**17**
17	Sterile margin distinct, fimbriate; basidiospore length/width < 4	***S.minutissima***
–	Sterile margin indistinct to almost absent; basidiospore length/width > 4	***S.tenuis***

## ﻿Discussion

*Sideraamericana* is discovered in USA and Canada, and the species is characterized by annual, resupinate basidiomata with silk sheen when dry, round pores (9–11 per mm), a dimitic hyphal system, and allantoid basidiospores measuring 3.5–4.2 × 1 μm. In our phylogeny, two specimens of *S.americana* form a lineage with strong support (99% BS, 100% BP, 1.00 BPP, Fig. 1). *S.americana* is closely related to *S.parallela* (98% BS, 100% BP, 1.00 BPP, Fig. 1), but basidiospores are longer in *S.americana* than in *S.parallela* (3.5–4.2 μm vs. 2.8–3.3 μm, Du et al. 2020). In addition, *S.parallela* has parallel tramal hyphae, while they are interwoven in *S.americana*.

*Sideraborealis* is discovered in boreal areas of China, including Gansu, Jilin, Qinghai, Shannxi, and Yunnan. The species is characterized by annual, resupinate basidiomata with cream to pinkish buff dry pore surface, angular pores (6–7 per mm), a dimitic hyphal system, and allantoid basidiospores measuring 3.9–4.1 × 1–1.1 μm. Phylogenetically, *S.borealis* clustered together with *S.vulgaris* with strong support (100% BS, 100% BP, 1.00 BPP, Fig. 1). Morphologically, *S.vulgaris* is different from *S.borealis* by the presence of capitate hyphal ends and “halocystidia” in tube mouths. Besides, basidiospores are longer in *S.borealis* than in *S.vulgaris* (3.9–4.1 μm vs. 2.9–3.6 μm, Niemelä and Dai 1997). *S.borealis* resembles *S.minutipora* by cream to buff fresh pores and similar pores (6–7 per mm vs. 5–7 per mm, Du et al. 2020). However, skeletal hyphae of *S.minutipora* become swollen in KOH, while they are unchanged in KOH in *S.borealis*. Besides, both species are distantly related (Fig. 1).

*Sideraamericana* and *S.borealis* are described from North China and North America; like most other species of *Sidera*, the two new species grow mostly on gymnosperm wood in temperate or boreal forests, but they are distinguished from existing species in the genus by morphology, geographic distribution and DNA sequences.

Boreal areas of China have the most important virgin forests in the country, and such forests provide favorable environments for some special wood-decaying fungi, e.g. *Heterobasidion* Bref., *Skeletocutis* Kotl. & Pouzar and *Sidera*, because fewer morphological characteristics existed among different species of each genus, and many species in the traditional definition are, in fact, the species complex. In recent years, the introduction of molecular systematics has greatly improved our understanding of the diversity of wood-rotting fungi in the boreal forests. Numerous new species have been found there (Dai et al. 2007, 2021; Yuan and Dai 2008; Tian et al. 2013; Li et al. 2014; Chen et al. 2015, 2016; Cui et al. 2019; Wang et al. 2021; Wu et al. 2021, 2022b), and we believe that more boreal new species will be found in the future.

## Supplementary Material

XML Treatment for Sidera americana

XML Treatment for Sidera borealis

XML Treatment for Sidera tibetica
